# Relationship between Tube Parameters and Corneal Endothelial Cell Damage after Ahmed Glaucoma Valve Implantation: A Comparative Study

**DOI:** 10.3390/jcm9082546

**Published:** 2020-08-06

**Authors:** Han Min Lee, Kyoung Nam Kim, Kee Sup Park, Nam Ho Lee, Sung Bok Lee, Chang-Sik Kim

**Affiliations:** 1 Department of Ophthalmology, Chungnam National University College of Medicine, 266 Munhwa-ro, Jung-gu, Daejeon 35015, Korea; lihanil12@naver.com (H.M.L.); red-mirr@hanmail.net (K.S.P.); sblee@cnu.ac.kr (S.B.L.); kcs61@cnu.ac.kr (C.-S.K.); 2 Department of Ophthalmology, Chungnam National University Hospital, 282 Munhwa-ro, Jung-gu, Daejeon 35015, Korea; 3 Mindeulle Eye Clinic, 9 Samsannam-ro, Boeun 28950, Korea; 74amg@naver.com

**Keywords:** Ahmed glaucoma valve implantation, anterior segment optical coherence tomography, corneal endothelial cell density

## Abstract

Purpose: We compared the clinical factors, including anterior chamber tube parameters, in patients with and without corneal endothelial cell damage after Ahmed glaucoma valve (AGV) implantation. Methods: In this retrospective and comparative case series, patients who underwent AGV implantation were enrolled consecutively. Serial specular microscopy was performed before and after AGV implantation. Patients were divided into two groups depending on whether there was a significant decrease in corneal endothelial cell density (ECD), which was determined by each patient’s rate of ECD change (%/year), calculated using linear regression analyses. Tube parameters such as the tube-cornea distance (TCD) and tube-cornea angle (TCA) were measured with anterior segment optical coherence tomography. Clinical factors related to the rate of ECD change were evaluated with regression analyses and compared between the two groups. The tipping point at which tube parameters became significantly associated with the rate of ECD change was identified with broken stick regression analyses. Results: There were 30 eyes (32.3%) with ECD damage (group 1) and 63 eyes (67.7%) without damage (group 2). The mean rate of ECD change (%/year) was −18.82 ± 22.97 and 2.14 ± 2.93 in groups 1 and 2, respectively (*p* < 0.001). The TCA was the only clinical factor associated with the rate of ECD change (regression coefficient, β = 1.254, *p* < 0.001). The tipping point in the TCA was 26.70° (95% confidence interval, CI: 23.75–29.64°). The mean TCD (mm) was 0.98 ± 0.38 and 1.26 ± 0.39 (*p* = 0.002), and the mean TCA (degrees) was 28.67 ± 7.79 and 36.35 ± 5.35 (*p* < 0.001) in groups 1 and 2, respectively. Conclusions: A wider TCA was protectively associated with the rate of ECD change, and the TCA was significantly narrower in patients with ECD damage. When inserting a tube into the anterior chamber, surgeons should therefore try to secure a wide TCA of about 30°. In patients with a narrow TCA after AGV implantation, increased attention should be directed toward whether ECD decreases continuously.

## 1. Introduction

Glaucoma drainage device (GDD) surgery has become a widely used option for treating medically uncontrolled glaucoma [1]. GDD surgery shows good results, including control of the postoperative intraocular pressure (IOP); additionally, the risk of complications is comparable to that for trabeculectomy [2,3,4,5]. Nevertheless, corneal endothelial damage remains a possible long-term complication of GDD surgery. Corneal endothelial cell density (ECD) is significantly decreased after GDD surgery [6,7,8,9,10,11]. Although the exact mechanism of ECD loss is not clear, researchers hypothesize that the relationship between the tube of the GDD and the cornea is one of the most important factors [6,11,12,13]. Kim and colleagues found an 11.5% decrease in the average ECD 6 months after surgery and a 15.3% decrease 12 months after surgery. Furthermore, the superior temporal cornea, which was closest to the tube, showed the largest decrease in ECD, 18.0% [10].

Recent reports have directly evaluated correlations between tube parameters measured by anterior segment optical coherence tomography (AS-OCT) and ECD damage [14,15,16,17]. They concluded that tubes positioned closer to the cornea led to an increased loss of adjacent ECD [16,17]. AS-OCT is a noncontact imaging modality that provides the detailed structure of the anterior part of the eyes, which can be used to measure several parameters related to angle and anterior chamber status [14,18,19].

To the best of our knowledge, studies on ECD damage after GDD surgery have analyzed patients as a single group, which may have resulted in an over- or underestimation of ECD damage. Overestimation means that ECD appears to inevitably decrease after GDD surgery. However, in clinical practice, it is not unusual for glaucoma patients to show no change in ECD after surgery. Underestimation means that in patients with ECD loss, the amount of damage is less because of the averaging effect.

Identifying differences in clinical factors in patients with and without ECD damage would be helpful in reducing the risk for ECD damage after GDD surgery. We therefore divided patients who underwent GDD surgery into two groups depending on their rate of ECD change (%/year): patients with ECD damage and patients without damage. We compared clinical factors, including tube parameters, between the two patient groups. In addition, we verified whether the tube parameters were related to the rate of ECD change and we identified tipping points that predicted the possibility of ECD damage after surgery.

## 2. Methods

### 2.1. Patients

This retrospective study was approved by the Institutional Review Board of Chungnam National University Hospital (IRB number: 2018-03-040). It was conducted in accordance with all relevant requirements of the Declaration of Helsinki. Patients with glaucoma who underwent Ahmed glaucoma valve (AGV) implantation (Model FP7 with a surface area of 184 mm^2^; New World Medical, Rancho Cucamonga, CA, USA) in our glaucoma clinic between January 2014 and June 2018 were consecutively enrolled.

All patients were followed for >12 months after surgery. Surgery was performed if any of the following criteria were met: intraocular pressure (IOP) > 21 mmHg and progressive optic nerve head damage, a defect in the retinal nerve fiber layer, or a defect in the visual field despite maximal tolerable medical or laser treatment. We excluded patients with congenital glaucoma, preoperative corneal decompensation, corneal endothelial dystrophy, including Fuchs dystrophy, posterior polymorphous dystrophy, or iridocorneal endothelial syndrome, or other corneal epithelial or stromal disorders, including scarring from previous corneal lacerations, which could have influenced the quality of specular microscopy. Patients who had previously undergone intraocular surgery, except for uncomplicated cataract surgery and trabeculectomy performed more than 6 months ago, were excluded. In addition, we excluded patients who had undergone any secondary intraocular surgery within 12 months of AGV implantation. If patients underwent secondary surgery more than 12 months after AGV implantation, data from before the surgery were collected. Electronic medical records were reviewed to assess various clinical factors, including age at surgery, sex, laterality of the operated eye, past medical history, glaucoma type, axial length, anterior chamber depth, IOP, results of preoperative and postoperative specular microscopic examinations, and follow-up months.

All surgeries were performed by a single glaucoma specialist (K.N.K). A fornix-based conjunctival flap was created in the superotemporal quadrant, and blunt dissection was performed between Tenon’s capsule and the episclera for AGV implantation. A half-thickness, rectangular, 3 × 5 mm, limbal-based scleral flap was created. Priming of the drainage tube was performed with a 27-gauge blunt cannula containing balanced salt solution (Alcon Laboratories, Fort Worth, TX, USA). In three patients, tube ligation was performed due to the very low opening pressure on tube priming [20,21]. Then, the AGV plate was inserted posteriorly into the sub-Tenon’s space, and two sutures anchored it to the sclera 8 mm posterior to the limbus. The anterior chamber was entered with a 23-gauge needle under the scleral flap, and the drainage tube was inserted through this entrance. The scleral flap was replaced and sutured to the posterior sclera. The conjunctiva and Tenon’s capsule were sutured back into their original positions. In all patients, 1% Isopto Atropine eye drops (Isopto Atropine; Alcon, Fort Worth, TX, USA) were used for 2 days after surgery, and 0.3% ofloxacin eye drops (Tarivid; Santen, Osaka, Japan) and 1.0% prednisolone acetate eye drops (Pred Forte; Allergan, Irvine, CA, USA) were used four times a day for 2 weeks and tapered over 6 months.

### 2.2. Corneal Specular Microscopy

Noncontact-type specular microscopy (Noncon Robo NSP-9900; Konan Medical, Tokyo, Japan) was performed before and after surgery. Routine examinations were performed 1, 3, 6, and 12 months after surgery. Specular microscopy was performed at intervals of 6–12 months. All examinations were conducted in five areas: the center, superior nasal, superior, superior temporal, and inferior areas of the cornea, while the patient fixated on a target. This modality automatically captured images of the endothelium once the patient fixated on the target. A “center-dot” method was used to measure ECD (cells/mm^2^). Details of this method are provided in our previous report [11].

### 2.3. Anterior Segment Optical Coherence Tomography

**Figure 1:**
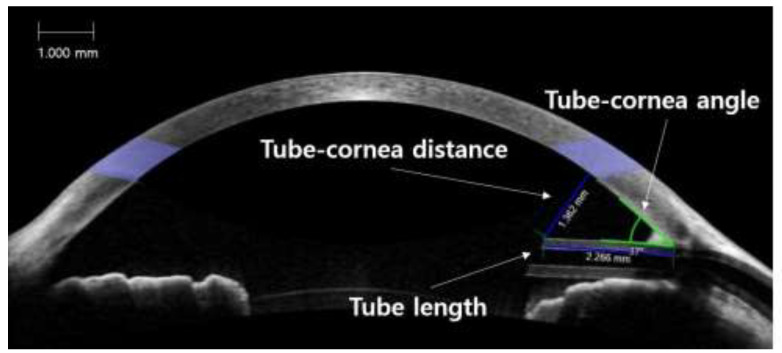
Anterior segment optical coherence tomography measurements of tube parameters: tube-cornea angle (TCA), tube-cornea distance (TCD), and tube length (TL).

All patients underwent anterior segment imaging using spectral-domain optical coherence tomography (Cirrus HD OCT; Carl Zeiss Meditec, Dublin, CA, USA) 3–6 months after surgery. During these examinations, the patients were asked to fixate on an internal fixation target. Images that lacked artifacts of eye motion and blinking were included in the analyses, and the best quality images were obtained with methods similar to those described by Hau et al. [22]. The scanning axis was positioned along the tube so that the position of the tube, including the tip in relation to the other structures of the anterior chamber, could be clearly visualized (Figure 1). The distance between the anterior tip of the tube and the cornea (TCD), perpendicular to the cornea, was measured. The length of the tube (TL) was measured from the point of entrance into the anterior chamber to the anterior tip of the tube. The tube-cornea angle (TCA) was defined as the angle between the posterior corneal surface and the anterior surface of the tube. Two investigators masked to other information relating to ECD (H.M.L and K.S.P) measured these tube parameters using an integrated ruler and angle indicator. The mean value of the two investigators was used as the measurement.

### 2.4. Statistical Analyses

PASW version 18.0 (SPSS, Chicago, IL, USA) was used for statistical analyses. Inter-rater agreement for tube parameter measurements on AS-OCT was assessed using the intraclass correlation coefficient (ICC). The rate of ECD change after AGV implantation was calculated using linear regression analyses (the slope of each regression equation represents the rate of ECD change in %/year). We calculated three rates of ECD change according to center area, superior temporal area, and the average value of five areas (the center, superior nasal, superior, superior temporal, and inferior areas). Patients were divided into two groups: those with ECD damage (group 1) and those without damage (group 2). ECD damage was defined as negative velocity in two or three of three rates of ECD change, or statistically significant negative velocity in any one of the three rates of ECD change. Group 2 contained other patients. Demographic variables and the rate of ECD change (%/year) were compared between the two groups with Student’s t-test, the chi-square test, or Fisher’s exact test. Student’s t-test was used to compare the AS-OCT measurements between the two groups. Univariate and multivariate regression analyses were used to identify variables associated with the annual ECD change (%). A scatter plot of tube parameters with mean ECD change was created. A broken stick nonlinear statistical model was fit to the data to calculate the tipping point at which tube parameters became significantly associated with the rate of ECD change. We estimated the tipping point using the Davies test conducted using R Language. *p*-value < 0.05 was assumed to indicate statistical significance.

## 3. Results

### Demographics

Data from 93 eyes of 91 patients who underwent AGV implantation were analyzed. There was no definite complication affecting the corneal endothelium, such as tube-corneal touch or collapse of the anterior chamber during or after surgery. There were 30 eyes with ECD damage (group 1; 24 males) and 63 eyes without ECD damage (group 2; 42 males). Mean age was 64.6 ± 11.6 years in group 1 and 60.4 ± 13.6 years in group 2 (*p* = 0.150). The mean ECD was 2194.03 ± 572.08 cells/mm^2^ in group 1 and 2324.19 ± 510.16 cells/mm^2^ in group 2 (*p* = 0.272). There were no significant differences between the two groups in sex, past medical history, axial length, anterior chamber depth, IOP, preoperative ECD (center, superior temporal), or follow-up period (all *p* > 0.05; Table 1).

Table 2 compares tube parameters obtained by AS-OCT between the two groups (Table 2). The ICCs of the TL, TCD, and TCA were 0.911, 0.907, and 0.925, respectively (all *p* < 0.001). The TL was 2.21 ± 0.70 mm in group 1 and 2.21 ± 0.69 mm in group 2 (*p* = 0.989). The TCD was significantly shorter in group 1 than in group 2 (0.98 ± 0.38 vs. 1.26 ± 0.39 mm, *p* = 0.002), and the TCA was significantly narrower in group 1 than in group 2 (28.67 ± 7.79° vs. 36.35 ± 5.35°, *p* < 0.001).

Table 3 compares the rate of ECD change according to center, superior temporal, and average values (the mean of five areas of the cornea) and the difference between the superior temporal and center areas. The reduction in average values was significantly faster in group 1 than in group 2 (−18.82 ± 22.97%/year vs. 2.14 ± 2.93%/year, respectively; *p* < 0.001). The reduction in the center and superior temporal areas was significantly faster in group 1 than in group 2 (all *p* < 0.001). In group 1, the rate of ECD change in the superior temporal area was −30.38 ± 26.18%/year, which was significantly more prominent than in the center area (*p* < 0.001).

Univariate regression analyses showed that the average rate of ECD change (%/year) was significantly associated with uveitic glaucoma, postoperative mean IOP, TCD, and TCA. We subjected parameters with *p* < 0.1 in univariate analyses to multivariate regression analyses, including sex, preoperative ECD, uveitic glaucoma, follow-up period, postoperative mean intraocular pressure, TCD, and TCA. The TCA was the only factor associated with the rate of ECD change (β = 1.254, *p* < 0.001; Table 4). There were strong positive correlations between any two variables among the center, superior temporal, and average value of preoperative ECD (correlation coefficients ≥ 0.843, all *p* < 0.001). For multivariate regression analyses, we selected the average ECD value as the preoperative ECD, with the expectation that it could represent overall ECD status.

**Figure 2:**
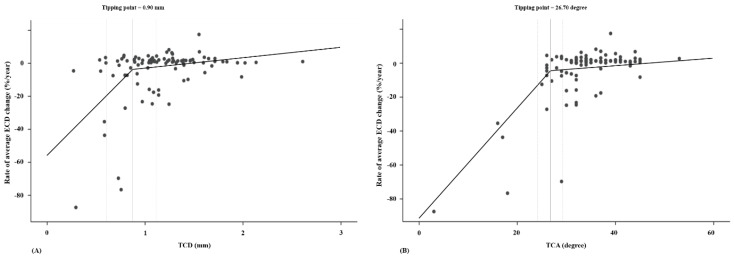
Scatter plots of the relationships between tube parameters and the rate of change in average endothelial cell density (ECD) in all patients: (**A**) tube-cornea distance (TCD), (**B**) tube-cornea angle (TCA). The tipping points of the TCD and TCA are 0.90 mm (95% confidence interval (CI): 0.68–1.11) and 26.70° (23.75–29.64°), respectively. The slopes for the rate of average ECD change as a TCD below and above the tipping point are 56.36 and 4.45, respectively (*p* = 0.003). The tipping point of the TCA location is not statistically significant (*p* = 0.366), and there is a significant difference between the rates below and above the slopes (3.75 and 0.57, respectively; *p* < 0.001).

Figure 2 shows the relationship between the rate of average ECD change and tube parameters in all patients. Estimates of the statistically optimal tipping points of the TCD and TCA were 0.90 mm (95% CI: 0.68–1.11) and 26.70° (23.75–29.64°), respectively. The TCD showed a tight CI around the deflection point, and the location of the tipping point was statistically significant (*p* = 0.042, Davies test). In patients with a TCD < 0.90 mm, the shorter the TCD, the faster the reduction in ECD (β = 56.36, 95% CI: 19.99–92.73). However, if the TCD was longer than 0.90 mm, the slope decreased (β = 4.45, 95% CI: −6.29–15.18). There was a significant difference between the two slopes associated with the rate of ECD change (*p* = 0.003). Similarly, in patients with a TCA < 26.70°, the narrower the TCA, the faster the reduction in ECD (β = 3.75, 95% CI: 2.83–4.67). However, if the TCA was wider than 26.70°, the slope decreased (β = 0.57, 95% CI: 0.12–1.03). Although the tipping point of the TCA location was not statistically significant (*p* = 0.366), there was a significant difference between the rates below and above the slopes (*p* < 0.001).

## 4. Discussion

According to our study results, 30 eyes (32.3%) showed ECD damage after AGV implantation, with a mean rate of ECD change of −18.82 ± 22.97%/year. However, there was no significant decrease in ECD in the other 63 eyes (67.7%). The rate of ECD change in patients without ECD damage was similar to the reduction in ECD in normal adults evaluated in a previous report [23]. The TCA and TCD were the clinical factors that were significantly different between the two patient groups with and without ECD damage. A wider TCA was the only protective factor associated with the rate of ECD change after multivariate regression analyses.

GDD surgery is useful for treating refractory glaucoma and is widely used [4,24,25]. Recently, there have been numerous reports of good surgical outcomes for GDD surgery, and not only has the frequency of glaucoma implant surgery increased significantly but its indications have also been extended [2,7,26,27,28]. However, corneal decompensation due to continuous ECD decrease is a leading cause of vision loss and is one of the most important and frequent complications of GDD surgery [4,7]. Reports of the frequency of corneal decompensation after GDD surgery with long-term (≥2 years) follow-up have ranged from 3.3% to 27% [6,7,11]. Lee et al. evaluated ECD changes in various locations of the cornea after AGV implantation and found that ECD decreased progressively up to 24 months after AGV implantation and that the change was significant compared to the baseline and control groups [10]. Kim et al. reported a decrease in central ECD after AGV implantation. In that study, patients without direct mechanical complications such as tube-corneal touch showed statistically significant ECD loss compared to baseline values at 2 years after surgery, and ECD decreased continuously up to 5 years after surgery, although without significance [11]. However, all previous studies have reported mean ECD reduction rates for the entire population of enrolled patients, which means that patients without ECD reduction after surgery were also included in these calculations. We hypothesized that ECD does not decrease in all patients after GDD surgery in practice. Therefore, we conducted a comparative study of patients with and without an ECD decrease.

Although many theories about the cause of ECD decreases have been proposed, including retrograde flow from the encapsulated reservoir to the anterior chamber, inflammation in the anterior chamber, intermittent tube-corneal contact, turbulence present at the tip of the tube, and a foreign body reaction to the tube material, the exact mechanism causing corneal endothelial damage after glaucoma tube shunt surgery remains unclear [6,7]. However, even in the absence of direct tube-corneal touch, the relationship between the tube and the cornea is expected to be an important factor regardless of the exact physiology. Previous findings on ECD loss (prominent in the superior temporal area) support this hypothesis. Lee et al. found a prominent ECD decrease in the superior temporal area (closest to the tube) [10]. Koo et al. reported that ECD was significantly lower in the superior temporal cornea (1611.9 ± 785.8) than either the central (1902.2 ± 841.4) or inferior nasal (1938.4 ± 823.5) cornea in patients with a GDD tube [16]. Similarly, in our study, annual loss was greater in the superior temporal area than in the central area in patients with ECD damage (−30.38 ± 26.18%/year vs. −17.82 ± 25.01%/year, *p* < 0.001). All tubes inserted in the anterior chamber had a bevel-up tip, which potentially directed turbulent flow toward the adjacent corneal endothelium. The superior temporal cornea would have been more affected by jet flow or turbulence than would other areas of the cornea. It has been postulated that the peripheral corneal endothelium contains progenitor cells that can act as a source of renewal for the center [29]. The very low proliferative capacity in the peripheral endothelium may be irreversibly affected by the presence of a tube nearby.

In previous reports, the distance from the tube to the posterior surface of the cornea was the most important of several tube parameters for decreasing the ECD [16,17,22]. Koo et al. found that positioning tubes closer to the cornea led to an increased loss of adjacent endothelial cells [16]. A recent prospective study by Tan et al. showed that a shorter TCD led to more ECD loss, which was most severe in the peripheral quadrant closest to the Baerveldt glaucoma drainage device tube [17]. Results of our multivariate analyses showed that of the AS-OCT parameters, only the TCA was a crucial factor in ECD decreases. Our results differ from previous studies that have reported that the TCD is the most important factor. However, the TCA and TCD are not independent variables: the TCD would be longer in patients with a relatively wide TCA than in those with a relatively narrow TCA. The TCD is also affected by the TL: the TCD would be longer in patients with a relatively long TL given the same TCA. In the present study, the TCD was shorter than in previous studies (1.1–1.7 mm) because the TL was also shorter than in previous studies [16,30,31]. We propose that the TCA is a more suitable reference target for lowering the risk for ECD damage, and the present study showed that few patients with ECD damage had a TCA above 29.64° (tipping point: 26.70°, 95% CI: 23.75–29.64). It also showed that patients had less ECD damage when the TCD was more than 1.11 mm (tipping point: 0.90 mm, 95% CI: 0.68–1.11). We suggest that it is important to secure a wider TCA to ensure a longer TCD when inserting the tube into the anterior chamber.

In multivariate regression analyses, the follow-up period had a borderline significant relationship with the rate of ECD change, with a longer period related to a slower ECD decrease. In the present study, the mean follow-up period was 28.3 ± 12.6 months (range: 12–71 months). We assume that this result was affected by specific cases. In three patients judged to have critical ECD decreases, the tube was removed or repositioned. Their follow-up periods for collecting data were relatively short, from 13 to 18 months. In addition, another patient with a very low preoperative ECD (mean value: 544 cells/mm^2^) did not show distinct cell patterns on specular microscopy after postoperative month 12, so data collection was terminated at that time. Multivariate analyses excluding these four cases showed no statistical significance between the follow-up period and the rate of ECD change (β = 0.139, *p* = 0.167).

This study was a retrospective study, so there are some limitations. First, the follow-up period and frequency of specular microscopy differed for each patient. However, we used linear regression analyses to calculate the rate of ECD change in each patient to reduce the limitation of the different examination periods. Second, AS-OCT was not performed repeatedly at the same time as postoperative specular microscopy. Recently, Park et al. reported that the silicone tube in the anterior chamber gradually moved toward the back of the cornea after AGV implantation [30]. Further prospective studies are needed to evaluate the relationship between tube position and ECD reduction over time. Third, although we used the same fixation target and the same examination of corneal endothelial cells at each examination, it is possible that we may not have examined identical areas of the cornea at every visit. Given the possible variability among tests, we used average values of ECD in the center, superior nasal, superior, superior temporal, and inferior scans as independent variables to calculate mean rate of ECD change. Finally, the TCA and TCD criteria for lowering the risk for ECD damage in this study are likely applicable when the tube is inserted into the anterior chamber and the TL is approximately 2.21 mm, but these criteria may differ if the tube is inserted into the sulcus or the TL is long.

In conclusion, we observed significant corneal ECD damage in 30 patients (32.3%) after AGV implantation. The TCA was significantly narrower and the TCD was shorter in patients with ECD damage. We suggest that it is possible to reduce the risk of ECD damage significantly by inserting the tube so that the TCA is greater than approximately 30°. Although glaucoma surgeons already try to position the tube as far from the posterior corneal surface as possible, according to previous reports and subjective experience, in some cases, the tube is placed with a narrow TCA unintentionally, due to poor cooperation or an extremely narrow palpebral fissure, or because of a persistently shallow anterior chamber due to short eyes or long-lasting hypotony. In patients with a narrow TCA and a resulting short TCD after surgery, we recommend frequent specular microscopy to detect persistent ECD damage as soon as possible, which is important in preventing irreversible vision loss caused by corneal decompensation after AGV implantation.

## Figures and Tables

**Table 1:** Characteristics of the patients.

Characteristics	Patients with ECD Damage (Group 1, *n* = 30)	Patients without ECD Damage (Group 2, *n* = 63)	*p*-Value
Age (years, mean ± SD)	64.60 ± 11.63	60.41 ± 13.58	0.150
Sex (M/F)	24/6	42/21	0.227
Laterality (R/L)	15/15	31/32	1.000
Systemic disease, *n* (%)			
DM	10 (33.33)	26 (41.27)	0.503
HTN	14 (46.67)	30 (47.62)	1.000
CVA	1 (3.33)	1 (1.59)	0.543
Axial length (mm)	24.58 ± 2.06	24.37 ± 2.12	0.641
Anterior chamber depth (mm)	4.06 ± 0.96	3.81 ± 0.86	0.238
Baseline ECD (cells/mm^2^)			
Average *	2194.03 ± 572.08	2324.19 ± 510.16	0.272
Center	2182.03 ± 569.97	2320.33 ± 500.55	0.237
Superior-temporal	2184.60 ± 574.20	2311.90 ± 532.75	0.296
Diagnosis, *n* (%)			0.275 ^†^
Neovascular glaucoma	10 (33.33)	30 (47.62)	
Primary open-angle glaucoma	9 (30.00)	11 (17.46)	
Uveitic glaucoma	5 (16.67)	11 (17.46)	
Chronic angle-closure glaucoma	3 (10.00)	1 (1.59)	
Pseudoexfoliation glaucoma	1 (3.33)	2 (3.17)	
Steroid induced glaucoma	1 (3.33)	1 (1.59)	
Other secondary glaucoma	1 (3.33)	7 (11.11)	
Preoperative IOP (mmHg)	36.63 ± 9.76	38.97 ± 11.76	0.234
Postoperative mean IOP (mmHg)	18.27 ± 4.25	19.13 ± 5.27	0.489
Final IOP (mmHg)	16.35 ± 3.45	15.71 ± 4.34	0.561
Follow-up period (months)	29.30 ± 14.67	27.75 ± 11.63	0.582
Postoperative topical CAI	15 (50.00)	35 (53.85)	0.661 ^†^

ECD = endothelial cell density; SD = standard deviation; DM = diabetes mellitus; HTN = hypertension; IOP = intraocular pressure; CAI = carbonic anhydrase inhibitor; CVA = cardiovascular accident. * Average value of the center, superior-nasal, superior, superior-temporal, and inferior area. ^†^ χ^2^ test.

**Table 2:** Tube parameters measured by anterior segment optical coherence tomography (AS-OCT).

Tube Parameters	Patients with ECD Damage (Group 1, *n* = 30)	Patients without ECD Damage (Group 2, *n* = 63)	*p*-Value
Tube length (mm)	2.21 ± 0.70	2.21 ± 0.69	0.989
Tube-cornea distance (mm)	0.98 ± 0.38	1.26 ± 0.39	0.002
Tube-cornea angle (degree)	28.67 ± 7.79	36.35 ± 5.35	<0.001

ECD = endothelial cell density. All values are expressed as the mean ± SD.

**Table 3:** Rate of postoperative change in corneal endothelial cell density (%/year).

Corneal Area	Patients with ECD Damage (Group 1, *n* = 30)	*p*-Value	Patients without ECD Damage (Group 2, *n* = 63)	*p*-Value	*p*-Value *
Average ^†^	−18.82 ± 22.97 (412.91 ± 503.96)	<0.001	2.14 ± 2.93 (49.74 ± 68.10)	0.342	<0.001
Center	−17.82 ± 25.01 (388.84 ± 545.73)	<0.001	1.95 ± 3.06 (45.25 ± 71.00)	0.612	<0.001
Superior temporal (ST)	−30.38 ± 26.18 (663.68 ± 571.93)	<0.001	2.56 ± 4.21 (59.18 ± 97.33)	0.440	<0.001
Difference between ST and center	−12.56 ± 17.01 (274.84 ± 371.50)	<0.001 ^‡^	0.61 ± 3.26 (14.10 ± 75.34)	0.145 ^‡^	<0.001

All values are expressed as the mean ± SD, percent value (absolute value). * *p*-value from an independent t-test between group 1 and group 2. ^†^ Average value of the center, superior-nasal, superior, superior temporal, and inferior areas. ^‡^ *p*-value results from a paired t-test between superior-temporal and the central change in corneal endothelial cell density (%/year).

**Table 4:** Univariate and multivariate regression analyses of clinical variables associated with the rate of postoperative change in average corneal endothelial cell density (%/year) *.

Characteristics	Univariate Analysis		Multivariate Analysis	
	β (95% CI)	*p*-Value	β (95% CI)	*p*-Value
Age (years, mean ± SD)	−0.067 (−0.324 to 0.190)	0.611		
Sex (male)	−6.329 (−13.599 to 0.941)	0.091	−4.506 (−10.274 to 1.262)	0.129
Laterality (right)	−0.577 (−7.282 to 6.128)	0.866		
Systemic disease				
DM	1.270 (−5.608 to 8.148)	0.718		
HTN	3.743 (−2.927 to 10.413)	0.274		
CVA	3.390 (−19.711 to 26.491)	0.774		
Axial length (mm)	−0.554 (−2.161 to 1.053)	0.501		
Anterior chamber depth (mm)	1.544 (−6.735 to 9.823)	0.716		
Baseline ECD (cells/mm^2^)				
Average *	0.006 (0.001 to 0.012)	0.068	0.003 (−0.001 to 0.007)	0.266
Center	0.006 (0.001 to 0.012)	0.059		
Superior temporal	0.006 (0.001 to 0.012)	0.074		
Diagnosis				
Neovascular glaucoma	4.710 (−1.991 to 11.411)	0.172		
Primary open-angle glaucoma	2.816 (−5.324 to 10.956)	0.499		
Uveitic glaucoma	−12.178 (−20.702 to −3.654)	0.006	−5.150 (−12.188 to 1.888)	0.155
Chronic angle-closure glaucoma	1.462 (−15.061 to 17.985)	0.863		
Pseudoexfoliation glaucoma	3.123 (−15.842 to 22.088)	0.748		
Steroid induced glaucoma	−3.738 (−26.837 to 19.361)	0.752		
Other secondary glaucoma	0.328 (−11.628 to 12.284)	0.957		
Preoperative IOP (mmHg)	0.104 (−0.180 to 0.388)	0.306		
Postoperative mean IOP (mmHg)	1.155(0.285 to 2.025)	0.011	0.547 (−0.163 to 1.257)	0.135
Final IOP (mmHg)	0.514 (−0.223 to 0.251)	0.467		
Follow-up (months)	0.252 (−0.011 to 0.515)	0.063	0.189 (−0.021 to 0.399)	0.081
Postoperative topical CAI	0.056 (−4.246 to 4.358)	0.542		
AS-OCT parameters				
Tube length (mm)	−2.088 (−6.949 to 2.773)	0.402		
Tube-cornea distance (mm)	14.493 (6.786 to 22.200)	<0.001	0.744 (−7.515 to 9.003)	0.860
Tube-cornea angle (degree)	1.427 (1.060 to 1.794)	<0.001	1.254 (0.776 to 1.732)	<0.001

CI = confidence interval; SD = standard deviation; DM = diabetes mellitus; HTN = hypertension; CVA = cardiovascular accident; CAI = carbonic anhydrase inhibitor. * Average value of the center, superior-nasal, superior, superior-temporal, and inferior areas.
